# Four and a half LIM domains protein 1 can be as a double-edged sword in cancer progression

**DOI:** 10.20892/j.issn.2095-3941.2019.0420

**Published:** 2020-05-15

**Authors:** Xiaofan Wei, Hongquan Zhang

**Affiliations:** ^1^Key Laboratory of Carcinogenesis and Translational Research (Ministry of Education), Department of Human Anatomy, Histology and Embryology, and State Key Laboratory of Natural and Biomimetic Drugs, Peking University Health Science Center, Beijing 100191, China

**Keywords:** Four and a half LIM protein 1 (FHL1), metastasis, tumor cell growth

## Abstract

Four and a half LIM domains protein 1 (FHL1), as the name suggests, contains four and a half LIM domains capable of interacting with various molecules, including structural proteins, kinases, and transcriptional machinery. FHL1 contains a zinc-finger domain and performs diverse roles in regulation of gene transcription, cytoarchitecture, cell proliferation, and signal transduction. Several studies have validated the importance of FHL1 in muscle development, myopathy, and cardiovascular diseases. Mutations in the FHL1 gene are associated with various myopathies. Recently, FHL1 was identified as a major host factor for chikungunya virus (CHIKV) infection in both humans and mice. Based on more recent findings over the last decade, FHL1 is proposed to play a dual role in cancer progression. On the one hand, FHL1 expression is suppressed in several cancer types, which correlates with increased metastatic disease and decreased survival. Moreover, FHL1 is reported to inhibit tumor cell growth and migration by associating with diverse signals, such as TGF-β and ER, and therefore considered a tumor suppressor. On the other hand, FHL1 can function as an oncogenic protein that promotes tumor progression upon phosphorylation, reflecting complex roles in cancer. This review primarily focuses on the dual role and underlying mechanisms of action of FHL1 in human cancer progression and its clinical relevance.

## Introduction

Four and a half LIM domain protein 1 (FHL1) belongs to the FHL protein family comprising FHL1, FHL2, FHL3, and FHL5 (activator of CREM in testis, ACT) in humans^1–3^. FHL4 was initially identified in mouse, and to date, no human ortholog has been reported^4^. FHL1, the founding member of the FHL protein family, was first discovered in skeletal muscle 23 years ago [initially designated “skeletal muscle LIM protein” (SLIM1)]^5^. Following the discovery of other highly homologous proteins, the term SLIM was replaced with FHL1^6–8^. All FHL proteins are characterized by a tandem arrangement of four complete LIM domains, and an N-terminal single zinc-finger domain with a consensus sequence identical to the C-terminal half of the LIM domain motif^9^. LIM domains mediate protein–protein interactions and are involved in linking proteins to both the actin cytoskeleton and transcriptional machinery^10^.

Apart from FHL5, FHL proteins are mainly expressed in muscle. FHL1 and FHL3 are most abundant in skeletal muscle whereas FHL2 is primarily expressed in cardiac muscle^11–13^. FHL5 is exclusively expressed in spermatids of adult testes. In the past decade, accumulating findings on the functions of FHL1 have been documented, among which FHL1-related myopathies are the most comprehensively characterized. Anomalies in the *FHL1* gene have been identified as the causative factor in various myopathies, such as X-linked myopathy^14^, muscular dystrophy, myofibrillar myopathy^15^, inflammatory myopathy^16^, reducing body myopathy^17^, and others^18^. In the majority of cases, skeletal muscle disorders are accompanied by cardiovascular diseases. FHL1 was recently reported to play a key role in cigarette smoking-induced chronic obstructive pulmonary disease^19^. In addition to the significant research progress regarding the function of FHL1 in skeletal muscle myopathies and cardiovascular diseases, studies on the involvement of FHL1 in cancer have grown exponentially. A number of comprehensive reviews have discussed the function of FHL1 in diverse tissues^1,18^, with specific focus on human myopathies^20^. Here, we present an overview of the cellular function of FHL1 and discuss recent advances in understanding its role in cancer.

## FHL1 domain structures and isoforms

### LIM domain

**Figure 1:**
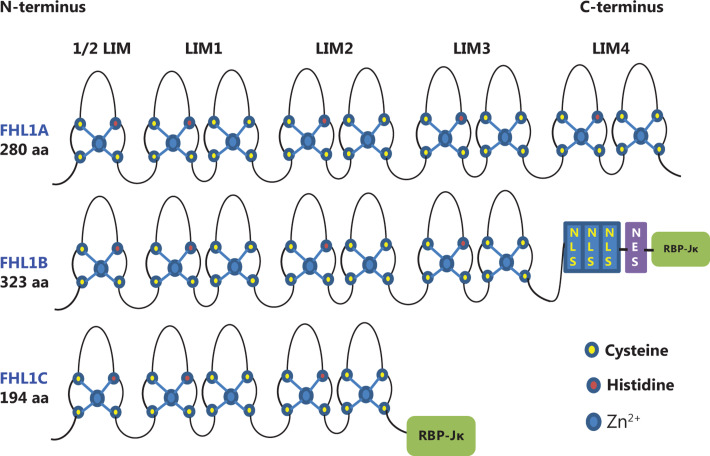
Structure and homology of FHL1 proteins. FHL1A contains four complete and an N-terminal half LIM domain. FHL1B contains the N-terminal three and a half LIM domains identical to FHL1A. However, in this case, the C-terminus is replaced by three nuclear localization signals (NLS), a nuclear export sequence (NES), and a binding site for RBP-Jκ. FHL1C comprises the N-terminal two and a half LIM domains identical to FHL1A and FHL1B, followed by a C-terminal RBP-Jκ-binding domain.

FHL1 is composed of an N-terminal half LIM and four complete LIM domains, and therefore classified as a LIM-only protein (**Figure 1**). Each LIM domain is separated by eight amino acid residues. To understand the mechanisms of action and biological implications of FHL1, clarification of the structure and function of the LIM domain is important. The LIM domain was initially identified in three homeodomain-containing proteins: (1) lin-11, which promotes asymmetric cell division in *C. elegans* during vulval development and regulation^21^, (2) Isl-1, which participates in the development of rat motor neurons^22^, and (3) Mec-3, which regulates specific *C. elegans* mechanosensory neurons^23^. The LIM acronym was derived from the first letters of LIN-11, ISL-1, and MEC-3. This domain has been identified in all eukaryotes examined, but is absent in prokaryotes^24^.

Structurally, the LIM domain is defined by the presence of a cysteine-rich double zinc-finger structure with the consensus sequence [C X2 C X16-23 H X2 (C/H) X2 C X2 C X16-23 C X2 (C/H/D)]^25^. The conserved cysteine and histidine residues coordinate the binding of two Zn^2+^ ions for every LIM domain, which is essential for stabilization of the individual zinc-finger structures. Earlier structural analyses have revealed a striking structural similarity between the C-terminal zinc finger of LIM domains and DNA-binding zinc finger of GATA. However, the zinc fingers of LIM domains do not directly bind DNA, instead mediating protein-protein interactions^26^. LIM domain-containing proteins function as molecular adaptor/scaffold proteins capable of interacting with other LIM domain proteins, tyrosine-containing motifs, PDZ domains, ankyrin repeats, and helix-loop-helix domains^27^. The presence of a LIM domain is emerging as a potential hallmark of proteins associated with both the actin cytoskeleton and transcriptional machinery. For instance, FHL proteins are able to localize to both the cytoskeleton to support the assembly of cytoskeletal and signaling complexes and the nucleus to function as either transcriptional coactivators or corepressors^25^.

### Alternate splice isoforms of FHL1

Full-length FHL1 generally refers to isoform A encoding a 32 kDa protein, also designated FHL1A/SLIM/SLIM1/KyoT/KyoT1. In addition to FHL1A, two isoforms, FHL1B (SLIMMER/KyoT3) and FHL1C (KyoT2), were initially identified in murine studies^28,29^. However, FHL1B and FHL1C do not exhibit the classical FHL protein structure (**Figure 1**). FHL1B is the larger isoform of FHL1, originally designated SLIMMER^8^, containing the first three and a half LIM domains found in FHL1 as well as a novel C-terminal sequence of 96 amino acids including three functional nuclear import sequences, a putative nuclear export sequence, and a region identical to the first putative sequence identified in FHL1C that binds RBP-Jκ. FHL1C is the shorter variant of FHL1 encoding a 22.0 kDa protein that is identical to FHL1 over the first two and a half N-terminal LIM domains but contains different protein sequences at the C-terminus, with a 27 residue putative RBP-Jκ binding region similar to that in FHL1B^30^. While both FHL1B and FHL1C are also expressed in skeletal and cardiac muscle, FHLA is the preponderant isoform in muscle. The three spliced variants exhibit distinct localization patterns, protein interaction partners, and functions. In this review, we focus on the function of FHL1 (FHL1A) in cancer.

## FHL1 expression and cellular localization

FHL1 is expressed in multiple tissues, with markedly high expression in skeletal and cardiac muscle and relatively low expression in several other organs, including colon, small intestine, placenta, ovary, prostate, testis, liver, spleen, and lung^2,5^. Interestingly, several studies have reported marked downregulation of FHL1 in various cancer types, including lung, prostate, breast, ovarian, colon, thyroid, brain, renal, oral, liver, and gastric cancer^31–36^, supported by microarray profiling and immunohistochemical analysis of human patient tissues.

At the subcellular level, FHL1 was initially shown to predominantly localize to the cytoplasm in association with focal adhesion and the actin cytoskeleton^8^. Subsequently, intracellular localization of FHL1 to the nucleus and stress fibers was documented^37^. FHL1 can additionally shuttle between these compartments in an integrin-dependent manner. In the nucleus, FHL1 is reported to bind numerous transcription factors, such as Smad4, RIP140, and oestrogen receptors, to affect gene transcription^38,39^. A recent study by our group^40^ focused on the mechanism underlying regulation of FHL1 localization and translocation into cells. The results showed that FHL1 is recruited to focal adhesion sites by the integrin-interacting protein, Kindlin-2, and translocates into the nucleus in a manner dependent on phosphorylation mediated by Src.

## Cellular functions and underlying mechanisms of action of FHL1

### Role of FHL1 in muscle cells

FHL1 is associated with multiple functions in both normal and tumor cells. Comprehensive studies on FHL1-related muscle cell function have been performed in view of the critical roles of FHL1 in myopathies. Transgenic experiments on mouse skeletal muscle have demonstrated that FHL1 induces muscle mass strength enhancement and atrophy^41,42^. FHL1 promotes myoblast adhesion, spreading, and migration in an integrin-dependent manner^37^. Similarly, overexpression of FHL1 induces myocyte elongation and may play an important role during the early stages of skeletal muscle differentiation through α5β1-integrin mediated signaling pathways^43^. A recent study showed that FHL1 regulates chicken myoblast differentiation and autophagy through interactions with LC3^44^. FHL1 silencing inhibited myoblast differentiation and expression of ATG5 and ATG7. Interactions between FHL1 with LC3 are reported to regulate correct autophagosome formation. Another report showed that FHL1 overexpression enhances migration and proliferation of primary human pulmonary artery smooth muscle cells (PASMCs)^45^. Interestingly, the group also showed that FHL1 colocalizes with Talin1 at focal adhesion sites in PASMCs, leading to the proposal that FHL1 alters the conformation of cytoskeletal proteins, such as talin, and plays a role in talin-mediated regulation of integrin signaling and cytoskeletal organization. Kubota and co-workers^46^ established a link between FHL1 and integrin. The group generated a knock-in mouse model with the same FHL1 mutation as human X-linked scapuloperoneal myopathy, one of the known FHL1-related diseases. In their study, 20 month-old mutant female mice showed signs of cardiomyopathy on echocardiograms, with increased systolic diameter and lower fractional shortening. Proteomic analyses indicated that abnormalities of the integrin signaling pathway (ISP) were associated with cardiac dysfunction, implicating ISP dysregulation in the pathogenesis of FHL1 myopathy^46^. Based on these findings, FHL1 is believed to be an important component of integrin signaling, although the precise mechanism remains to be elucidated. Moreover, FHL1 appears to exert positive effects on cellular functions, including growth and migration, in normal cells. 

### Role of FHL1 in virus infection

Recent studies have identified FHL1 as a major host factor for chikungunya virus (CHIKV) infection in both humans and mice^47,48^. The potential host genes required for CHIKV infection were determined *via* genome-wide CRISPR–Cas9 screening of human HAP1 cells, among which FHL1 showed the most significant enrichment. Consistent with this finding, knockdown of FHL1 markedly inhibited CHIKV21 infection and release of infectious particles. Conversely, overexpression of FHL1 enhanced CHIKV infection in BeWo and HepG2 cells that generally show low susceptibility to CHIKV infection. Further infection studies revealed resistance of primary dermal fibroblasts and muscle cells from patients with FHL1 deficiency to CHIKV infection. Molecular mechanism analyses disclosed that direct interactions between FHL1A and CHIKV nsP3, which contains a hypervariable domain that regulates RNA amplification, are crucial for the proviral function of FHL1.

## Role of FHL1 in cancer progression

### FHL1 suppresses tumor cell growth

**Figure 2:**
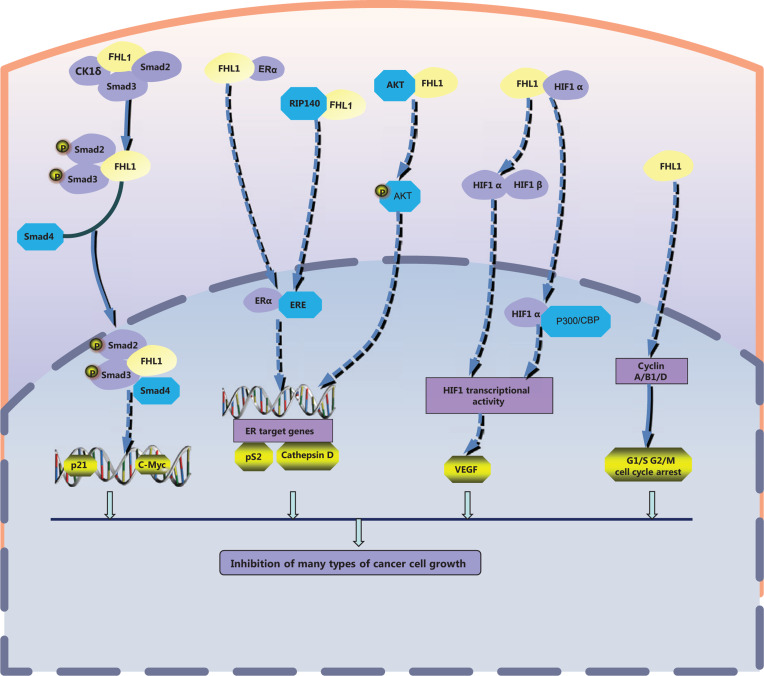
FHL1 functions as a tumor suppressor. FHL1 interacts with Smad2, Smad3, and CK1δ in hepatocellular carcinoma cells, and enhances phosphorylation of Smad proteins and nuclear translocation of Smad4 to regulate downstream target genes, such as p21 and c-myc. In human breast cancer cells, FHL1 interacts directly with ERα or inhibits AKT to decrease transcriptional activity of ERα, resulting in suppression of estrogen target gene expression, including pS2 and cathepsin D52. Furthermore, RIP140 cooperates with ER to inhibit estrogen signaling and estrogen-responsive target gene transcription. FHL1 inhibits HIF-1 transcriptional activity by competing with HIF-1 for binding to the co-activators, p300/CBP48, or blocking HIF1α-HIF1β heterodimerization. Levels of G1 and G2/M phase-related proteins, including cyclin A, B1, D, and E, are suppressed by FHL1 to mediate G1 and G2/M cell cycle arrest in lung cancer cells.

FHL1 appears to execute opposite functions in tumor and normal cells. The protein was initially identified as a tumor suppressor with inhibitory effects on cell growth and migration (**Figure 2**). A preliminary report from twelve years ago demonstrated Src tyrosine kinase-mediated suppression of FHL1 expression through phosphorylation of the focal adhesion adaptor protein, Crk-associated substrate (Cas), in v-Src-transformed cells that resulted in promotion of tumor cell growth^31^. Thus, FHL1 is considered a tumor suppressor gene that acts downstream of v-Src and Cas to specifically inhibit anchorage-independent cell growth and migration. Consistently, the group showed that v-Src blocked FHL1 expression independent of MAPK activity and v-Src mediated methylation of the promoter region of the FHL1 gene in transformed cells^49^. Asada et al.^50^ identified *FHL1* as a tumor suppressor gene on chromosome X inactivated by promoter methylation in gastrointestinal cancer. Consistently, methylation-silencing of FHL1 has been detected in multiple gastric and colon cancer cell lines and surgical gastrointestinal cancer specimens. Gain- and loss-of-function experiments clearly indicate that FHL1 inhibits gastrointestinal cancer cell growth, migration, and invasion^50^. Analogous to these findings, *FHL1* gene silencing through CpG hypermethylation is reported to promote proliferation, migration, and invasion activities of human bladder cancer cells^51^.

FHL1 additionally inhibits anchorage-dependent and -independent growth of human hepatocellular carcinoma cells, both *in vitro* and *in vivo*. FHL1-dependent decrease in cancer cell growth is mediated by interactions with SMAD proteins, leading to a transforming growth factor (TGF)-like response^52^. FHL1 physically and functionally interacts with Smad2, Smad3, and Smad4 in a TGF-β–independent manner. Through casein kinase 1 (CK1δ), FHL1 enhances phosphorylation of Smad proteins and promotes nuclear translocation of Smad4 to stimulate the tumor suppressor gene, p21, and suppresses the oncogene, c-myc. Thus, CK1δ and Smad4 are required for FHL1-mediated inhibition of hepatocellular carcinoma (HCC) cell growth. An investigation focusing on the regulation of FHL1 in hypoxia-inducible factor 1 (HIF-1) activity presented another potential mechanism through which FHL1 participates in liver cancer progression. This study reported that FHL1 inhibits HIF-1 transcriptional activity by competing with HIF-1 for binding to the co-activators p300/CBP^53^. Subsequently, FHL1 was shown to block HIF1α-HIF1β heterodimerization for regulation of HIF1 activity in hepatocellular carcinoma cells, leading to decreased promoter activity and expression of vascular endothelial growth factor (VEGF), an important target gene of HIF1α^54^. Another study showed that FHL1 and Smad4 synergistically inhibit promoter activity and expression of VEGF in HepG2 hepatoma cells^39^. FHL1 methylation is additionally involved in the associated mechanisms in human liver cancer. Furthermore, miR-410 may function as an oncomiR in liver and colorectal tumors by specifically targeting the 3′ UTR of FHL1 and promoting its methylation^55^. A recent epigenetic analysis identified FHL1 as a tumor suppressor gene in human liver cancer and indicated that EZH2-imediated H3K27me3 is involved in epigenetic repression of FHL1 in HCC^56^.

Similarly, FHL1 appears to play a suppressive role in breast cancer through interactions with multiple signaling pathways. FHL1 inhibits both anchorage-dependent and -independent breast cancer cell growth, which could be mediated by physical and functional interactions with estrogen receptors (ER) and modulation of transcriptional activities of ERα and ERβ. FHL1 acts as a negative regulator of estrogen-responsive transcription, resulting in decreased expression of estrogen target genes, including pS2 and cathepsin D^57^. Additionally, FHL1 binds the 140 kDa receptor-interacting protein (RIP140), leading to inhibition of transcriptional activity of ER. Upon co-expression, FHL1 and RIP140 synergistically inhibit estrogen signaling and estrogen-responsive target gene transcription^38^. Another report suggests that FHL1 suppresses the transcriptional activity of ERα in human breast cancer cells through regulation of AKT^58^. FHL1 interacts with AKT and inhibits its phosphorylation in response to insulin, resulting in repression of estrogen receptor-responsive gene transcription. The collective findings established an association between the effects of FHL1 on the estrogen signaling pathway and breast cancer cell growth.

A suppressive role of FHL1 in tumor cell growth associated with the cell cycle has also been documented. For instance, FHL1 has been shown to inhibit both anchorage-dependent and -independent growth of human lung cancer cells *via* cell cycle arrest. FHL1 promoted cell cycle arrest at both G1 and G2/M in lung cancer cells, thus regulating G1 and G2/M phase-related proteins, including significant inhibition of cyclin A, cyclin B1, and cyclin D, as well as upregulation of the cyclin dependent kinase inhibitors, p21 and p27^59^. Another study showed that FHL1 inhibits growth of tongue squamous cell carcinoma cells by inducing G1/S cell cycle arrest, potentially through suppression of cyclin D and cyclin E expression^60^.

### FHL1 regulates tumor metastasis

A study by Sakashita et al.^61^ documented the clinical significance of FHL1 expression in gastric cancer. Their results showed that tumors expressing low levels of FHL1 displayed deeper invasive ability into the serosal layer. Lower FHL1 expression was significantly correlated with frequent incidence of distant metastasis. Consequently, a correlation between FHL1 expression in tumor tissue and invasion and metastasis of gastric cancer was established by another group^62^. Lower FHL1 expression was positively associated with lower degree of differentiation, higher lymph node metastasis, and greater invasive potential of gastric cancer. The effects of FHL1 on the invasion and metastasis of gastric cancer were further confirmed *in vitro*. Overexpression of FHL1 in the human gastric cancer cell line, MKN45, significantly inhibited invasiveness and metastatic ability, as determined using the Transwell assay. Reduced FHL1 expression may thus contribute to enhanced tumor aggressiveness, correlating with significantly shorter survival in primary gastric cancer patients^61,62^.

Similarly, association of FHL1 expression with esophageal squamous cell carcinoma (ESCC) progression was reported^63^. FHL1 expression was markedly reduced in 82 human ESCC tissues, compared with matched adjacent normal esophageal tissues. Positive FHL1 expression was significantly correlated with differentiation, lower lymph node metastasis, early T stage, and clinical stage. Correspondingly, patients with negative FHL1 expression in ESCC displayed significantly shorter overall survival than those positive for FHL1 expression. Results from this study support the utility of FHL1 expression as an independent prognostic factor for esophageal cancer.

Recently, FHL1 involvement in the mechanism of long intergenic noncoding RNA 00261 (LINC00261) was reported, indicating a tumor suppressor role in non-small cell lung cancer (NSCLC)^64^. Significant downregulation of LINC00261 in tumor samples was correlated with lymphatic metastasis, tumor size, tumor stage, and patient survival. MiR-105 was further identified as a direct target of LINC00261 and FHL1 as a novel downstream target of miR-105. LINC00261 suppressed metastasis and proliferation of NSCLC cells through suppressing miR-105 and upregulating FHL1 expression *in vitro* and *in vivo*.

Although suppressive effects of FHL1 on metastasis are reported in several cancer types, no correlation of FHL1 with metastasis or invasion has been detected in human oral cancers^65^, including tongue squamous cell carcinoma (TSCC)^60^. Significant downregulation of FHL1 was demonstrated in all oral squamous cell carcinoma (OSCC)-derived cell lines and tissues from human patients, mediated by CpG hypermethylation of the FHL1 promoter region. A study on the correlation between FHL1 and clinical classification in OSCCs in 59 patients revealed no significant differences in expression of FHL1 in relation to clinicopathologic features, including primary tumor size, differentiation, and node metastasis. Likewise, although FHL1 inhibited anchorage-dependent and -independent growth of TSCC cells *in vitro* and tumor growth in nude mice, no effects were evident on migration, invasion, and metastasis, leading to the conclusion that FHL1 plays an important role in development but not progression of TSCC. The reasons underlying the distinct cellular functions of FHL1 in digestive and oral cancers require further investigation. One possible explanation is that FHL1 induces different modifications in diverse cell types, resulting in multiple effects. Cellular functions of FHL1 may be cell type- and tissue-dependent.

### Promotory effects of FHL1 in tumor cells

FHL1 is a well established tumor suppressor and several reports have confirmed its inhibitory effects on cell growth, migration, invasion, and metastasis. Downregulation of FHL1 expression is significantly associated with poorer overall survival (OS) in various cancer types, including gastric cancer, head-and-neck squamous cell carcinoma^66^, and esophageal squamous cell carcinoma. However, recent investigations have uncovered an opposite role of FHL1 in tumors.

While FHL1 is reported to inhibit human liver cancer, a study by Zhou et al.^67^ showed that FHL1 could effectively promote paclitaxel resistance in HCC cells, both *in vitro* and *in vivo*, through inhibition of caspase-3. FHL1 enhanced anchorage-independent growth of HCC cells and tumor growth in nude mice treated with paclitaxel. Knockdown of FHL1 in HCC cells rendered the cells more sensitive to paclitaxel than oxaliplatin. Accordingly, the authors suggested that FHL1 presents a promising molecular target for HCC therapy. Inhibition of FHL1 function could be explored as a potential therapeutic strategy to increase the anticancer activity of the drug.

Xu and co-workers^68^ demonstrated that FHL1 induces radiotherapy resistance in breast and cervical cancers. In a Kaplan-Meier survival analysis, higher FHL1 expression in tumors of breast cancer patients that had received radiotherapy was associated with poorer disease-free survival (DFS) and OS. Interestingly, no significant differences in DFS and OS of non-treated breast cancer patients were observed between groups with high and low FHL1 expression. Consistently, knockdown of FHL1 *in vitro* increased the sensitivity of cancer cells to ionizing radiation (IR). FHL1 knockdown cancer cells or FHL1 KO MEFs displayed shorter survival upon exposure to IR. Mechanistic experiments revealed that FHL1 promotes CDC25 phosphorylation by forming a complex with CHK2 and CDC25 and blocking interactions between the two proteins. FHL1 induced sequestration of CDC25C in the cytoplasm through binding to 14-3-3 protein, thereby inhibiting CDC25C activity. Furthermore, FHL1 increased IR-induced G2/M arrest through regulation of CDC25C, finally leading to radiotherapy resistance. This study further identified an 11 amino acid motif within the LIM domain (eLIM) that interacts with CHK2/CDC25C, blocks CHK2-CDC25 and CDC25/14-3-3 interactions, enhances CDC25 activity and consequently, promotes cancer radiosensitivity *via* mitotic catastrophe and apoptosis. The collective results indicate that inhibition of LIM protein or use of eLIM may present novel strategies for improving tumor response to radiotherapy.

**Figure 3:**
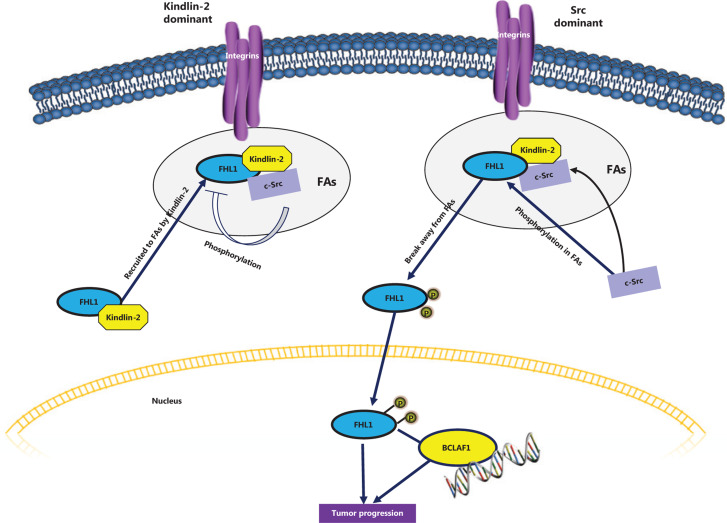
Phosphorylated FHL1 functions as a tumor promoter. Src interacts with and phosphorylates FHL1, which immediately trigrers translocation into the nucleus. Phosphorylated FHL1 binds to and cooperates with nucelar transcription factor BCL, promoting tumor cell growth. However, in the presence of excessive cellular Kindlin-2, FHL1 is recruited to focal adhesion sites and forms a complex with Kindlin-2 and Src. Interactions between Src and FHL1 and subsequent Src-induced phosphorylation of FHL1 are suppressed in this state, which maintains the tumor suppressive role of FHL1.

A recent study by our group demonstrated a tumorigenic effect of FHL1 upon phosphorylation by the tyrosine kinase Src^40^. Specifically, Src phosphorylates the protein at two tyrosine residues, Y149 and Y272. Following phosphorylation, FHL1 translocates into the nucleus where it binds the transcription factor BCLAF1. The effects of different nutants of FHL1 on lung cancer cell proliferation were additionally investigated. Consistent with previous reports, wild-type FHL1 repressed cell growth and migration *in vitro* and a nonphosphorylatable mutant of FHL1 exerted an even stronger inhibitory effect on cell growth and migration. Conversely, a phosphomimetic mutant of FHL1 promoted proliferation and migration. When cells expressing different FHL1 mutants were injected into mice, similar effects were observed* in vivo*. Cells expressing phosphomimetic FHL1 grew faster and displayed larger tumors than those expressing wild-type or nonphosphorylatable FHL1. Furthermore, phosphorylated FHL1-stimulated tumor cell growth appeared dependent on BCLAF1. Knockdown of BCLAF1 blocked phospho-FHL1-mediated tumor growth. Importantly, FHL1 phosphorylation was elevated in tissues obtained from various human cancer types, including lung, liver, gastric, rectal, and esophageal cancers, although total FHL1 levels were decreased in these tumors. Interestingly, the integrin-binding protein, Kindlin-2, bound to and recruited FHL1 to form a tripartite complex with Src at focal adhesion sites. Furthermore, Kindlin-2 competed with Src to interact with FHL1. Under conditions of overexpression of Kindlin-2 in cells, both interactions between FHL1 and Src and FHL1 phosphorylation were suppressed and the tumor suppressor function of FHL1 was maintained. The results suggest that the role of FHL1 in tumor progression is dependent on the relative levels of Kindlin-2 and Src activity in cells, which determine whether FHL1 is anchored at focal adhesion sites and where it inhibits tumorigenesis. Upon phosphorylation, FHL1 translocates to the nucleus where it acts as a tumor promoter (**Figure 3**).

In addition to the above investigations, a recent publication reported complex functions of FHL1 in tumors^69^. Fu and colleagues^69^ demonstrated that FHL1 is a novel and powerful prognostic indicator and potential therapeutic target in acute myeloid leukemia (AML), including non-acute promyelocytic leukemia (non-APL) AML and cytogenetically normal AML (CN-AML), by comparing genome-wide expression and clinical data from three independent AML datasets. Analysis of 1,298 adult patients with *de novo* AML and 407 CN-AML patients revealed high expression of FHL1 in association with poorer overall, event-free, and relapse-free survival rates. FHL1 was further identified as an independent predictor of poor outcomes in AML when combined with prognosis-related clinical factors and genetic abnormalities, such as MLL-PTD, TP53, and RUNX1 mutations. Gene Set Enrichment Analysis performed to determine the biological functions related to FHL1 and potential molecular mechanisms showed enrichment of FHL1-related genes in leukemia stem cell (LSC) signatures, tumor-associated signaling pathways, and transmembrane transport of chemotherapeutic drugs. Furthermore, FHL1 was highly expressed in LSCs and its knockdown enhanced the sensitivity of AML cells to cytarabine *in vitro*, supporting the involvement of FHL1 in chemotherapy resistance and relapse of AML. In view of these results, it is proposed that FHL1 is a strong independent indicator of prognosis for AML patient and FHL1-targeted intervention may present a novel and effective strategy for AML treatment. A pertinent issue that remains to be resolved is whether FHL1 undergoes a specific type of posttranslational modification (PTM), such as phosphorylation, in AML that promotes oncogenic activity.

## Conclusions and perspectives

FHL1 belongs to the LIM-only protein family characterized by four and a half highly conserved LIM domains. The protein is involved in multiple cellular activities including cytoskeletal remodeling, biomechanical stress response, cell growth, and transcriptional regulation. The diverse functions of FHL1 may be mediated by multiple binding partners linked to various signaling pathways, such as integrin, TGF-β, MAPK, and ER. FHL1 performs important roles in muscle development and human diseases, especially myopathies. Mutations in the *FHL1* gene have been identified as the cause of several skeletal muscle diseases.

The significant involvement of FHL1 in cancer pathways has become apparent over the last decade. FHL1 is widely downregulated in various cancers, mainly through gene silencing owing to CpG hypermethylation. Low FHL1 expression is markedly correlated with stronger metastasis and decreased survival in some cancer types. Inhibitory effects of FHL1 on anchorage-dependent and -independent cell growth and migration have been demonstrated in various tumor cell lines, leading to the inference that FHL1 is a tumor suppressor. However, more recent experiments have revealed that FHL1 induces radiation resistance in cancer cells, and increased expression is associated with significantly poorer disease-free and overall survival rates in breast cancer patients subjected to radiotherapy. Another recent study by our group implicated phosphorylated FHL1 induced by Src in tumor cell growth and migration. **Table 1** summarizes the known FHL1 protein interactions in cancer progression. The roles and mechanisms of action of FHL1 in cancer progression are more complex and diverse than previously understood and require further investigation.

At present, the PTMs of FHL1 are not fully understood. In view of our finding that phosphorylation of FHL1 dramatically alters the role of FHL1 from tumor suppressor to promoter, it is worth investigating whether other types of PTM, such as acetylation and ubquitination, regulate subcellular location and cellular function of FHL1. Perhaps the most promising area of future study is elucidation of the precise roles and regulatory mechanisms of FHL1 in both nuclear and cytoplasmic compartments, which should offer novel insights into how aberrant FHL1 expression and modification patterns influence tumor progression and provide new avenues for therapeutic intervention.

## Figures and Tables

**Table 1:** FHL1-interacting proteins involved in cancer progression

Protein	Interaction domain	Function	Reference
Smad2	n.d.	FHL increases Smad2/3 phosphorylation, enhances interactions of Smad2/3 and Smad4 in a CK1δ-depedent manner	^52^
Smad3	n.d.		^52^
Smad4	n.d.		^39,52^
Casein kinase 1, delta (CK1δ)	n.d.		^52^
Receptor interacting protein of 140 kDa (RIP140)	All domains of FHL1	FHL1 and RIP140 synergistically regulate transcription of estrogen signaling	^38^
Estrogen receptor α (ERα)	ERα (1–185) fragment containing the AF1 domain; LIM domains 1, 2 and 3	FHL1 inhibits the transcriptional activities of ERα and ERβ	^54^
Estrogen receptor β (ERβ)	1–145 aa of ERβ containing N-terminal AF1 domain		^54^
AKT	n.d.	FHL1 inhibits ERα activity through repression of AKT phosphorylation	^58^
p300	n.d.	FHL1 binds to p300/CBP, disrupting binding with HIF-1α	^53^
CBP	n.d.		^53^
HIF1α	HIF1α region containing basic helix-loop-helix (bHLH) motif and PER-ARNT-SIM domain; A single LIM domain	FHL1-3 inhibits HIF1α -dependent VEGF promoter activity and VEGF expression	^54^
CHK2	CHK2 (220–356 aa) containing the N-terminal portion of the protein kinase domain, an 11 aa motif, namely W/FHwwCFwCwwC (eLIM)	FHL1 inhibits CDC25 phosphorylation by forming a complex with CHK2 and CDC25, and sequesters CDC25 in the cytoplasm through interactions with 14-3-3	^68^
CDC25	CDC25C (328 and 383 aa) containing a partial catalytic domain; eLIM		^68^
14-3-3	(100–255 aa) of 14-3-3 containing the target binding pocket		^68^
Src	Kinase domain of Src; LIM4 domain	Src phosphorylates FHL1, leading to a switch in activity from tumor suppressor to promoter. Kindlin-2 competes with Src for binding to FHL1	^40^
Kindlin-2	FERM domain of kindlin-2; LIM4 domain		^40^
